# Best Foot Forward: Nanopore Long Reads, Hybrid Meta-Assembly, and Haplotig Purging Optimizes the First Genome Assembly for the Southern Hemisphere Blacklip Abalone (*Haliotis rubra*)

**DOI:** 10.3389/fgene.2019.00889

**Published:** 2019-09-25

**Authors:** Han Ming Gan, Mun Hua Tan, Christopher M. Austin, Craig D. H. Sherman, Yen Ting Wong, Jan Strugnell, Mark Gervis, Luke McPherson, Adam D. Miller

**Affiliations:** ^1^Centre for Integrative Ecology, School of Life and Environmental Sciences, Deakin University, Geelong, VIC, Australia; ^2^Deakin Genomics Centre, Deakin University, Geelong, VIC, Australia; ^3^Centre for Sustainable Tropical Fisheries and Aquaculture, James Cook University, Townsville, QLD, Australia; ^4^Southern Ocean Mariculture, Port Fairy, VIC, Australia; ^5^Jade Tiger Abalone, Indented Head, VIC, Australia

**Keywords:** abalone, Oxford Nanopore, hybrid assembly, heterozygosity, *Haliotis*, heat shock protein 70

## Introduction

**Figure 1:**
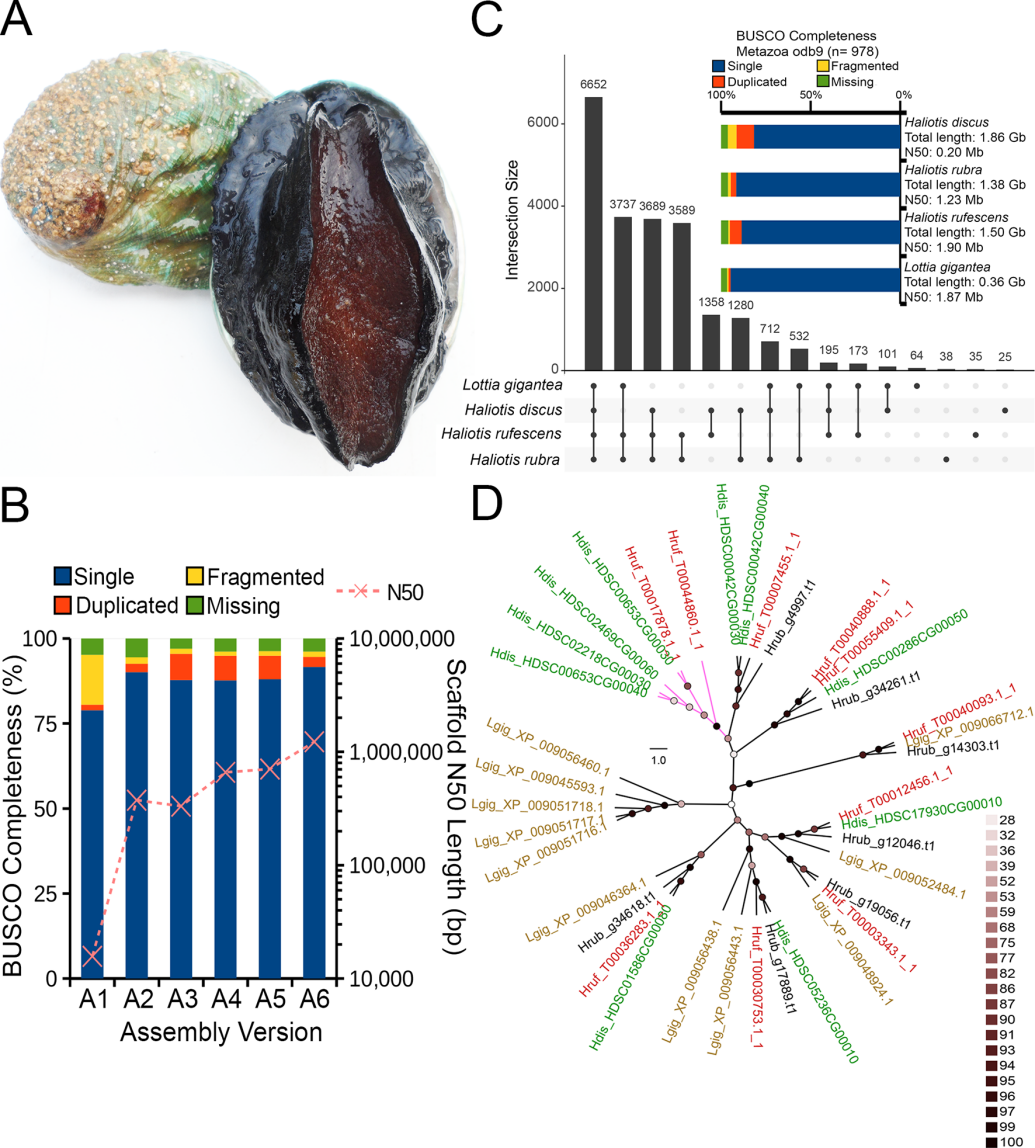
**(A)** The blacklip abalone (*Haliotis rubra*). Photo by Mark Gervis. **(B)** Scaffold N_50_ length and BUSCO genome assessment after each assembly iteration (see **Table 1** for additional details). **(C)** UpSet plot showing unique and shared protein ortholog clusters across the four marine gastropod genomes. Connected dots represent the intersections of overlapping orthologs with the vertical black bars above showing the number of orthogroups in each intersection. **(D)** IQTree Maximum likelihood tree depicting the evolutionary relationships among HSP70 proteins. The tree was mid-point rooted, and nodes were colored according to the ultrafast bootstrap support values. Scale bar represents number of substitutions *per* site. Tip labels were colored according to species name, and clade without *H. rubra* was colored purple. Four-letter code preceding each protein accession indicates species name. Hdis, *H. discus hannai*; Hrub, *H. rubra*; Hruf, *H. rufescens*; Lgig, *L. gigantea*.

Marine molluscs of the family Haliotidae, commonly referred to as abalone, are a group of benthic reef species targeted by commercial fisheries in 11 countries, forming an important global industry worth approximately US $180 million (Gordon and Cook, 2013). Many abalone fisheries have collapsed in recent decades due to overexploitation, environmental change, and disease, with a number of target species now listed as endangered or considered “species of concern” (Hauck and Sweijd, 1999; Leiva and Castilla, 2002; Gruenthal and Burton, 2005; Kashiwada and Taniguchi, 2007). The world’s largest thriving abalone fisheries persist in southern Australia which is currently home to a thriving and rapidly growing aquaculture industry making up approximately 10% of Australia’s export market. In this region, the abalone species *Haliotis rubra* (**Figure 1A**) was targeted in five states extending from Western Australia to southern New South Wales and Tasmania with a net value of US $79 million (Mundy et al., 2014).

To keep up with global demand and to counter disease and environmental stress, effective management of wild stock and farmed abalone is vital to ensure maximized productivity, sustainability, and biosecurity of the industry. To date, population and quantitative genetic research have contributed genomic resources to assist *H. rubra* fisheries management and conservation (Baranski et al., 2008; Miller et al., 2016; Kijas et al., 2019); however, a fully assembled and annotated genome sequence for this species is still unavailable. Such a resource is needed to assist future selective breeding programs, environmental stress and adaptation research, and fundamental genomic and evolutionary studies geared toward bolstering *H. rubra* fisheries and aquaculture.

Recent years have seen an increased supplementation of genome assemblies with PacBio and/or Nanopore long read sequences which often led to a substantial improvement in the genome contiguity and scaffold/contig reduction (Austin et al., 2017; Daccord et al., 2017; Zimin et al., 2017; Tan et al., 2018). For example, hybrid assembly of the clownfish genome with 11× Nanopore and 54× Illumina reads generated 94% fewer scaffolds and 18-fold longer N_50_ length compared to the Illumina-only assembly (Tan et al., 2018). To date, two abalone genomes are publicly available and were both assembled using PacBio and Illumina reads (Nam et al., 2017; Masonbrink et al., 2019). The first abalone genome belonging to the species *Haliotis discus hannai* was reported 2 years ago with a scaffold N_50_ of 211 kb (Nam et al., 2017). Two years later, the second abalone genome (*H. rufescens*) was published with a substantially improved contiguity (pre- and post-HiRise2 scaffold N_50_ of 588 kb and 1.895 Mb, respectively) presumably due the availability of higher PacBio data coverage and the use of a dedicated MaSuRCA hybrid assembler (Zimin et al., 2017; Masonbrink et al., 2019).

It was recently demonstrated that contiguous and accurate *de novo* assembly of metazoan genomes can be obtained with modest long read coverage (<30× coverage) by merging long-read only assembly with hybrid assembly (Chakraborty et al., 2016). However, this meta-assembly approach has not been extensively tested on heterozygous genome assemblies where a large proportion of the assemblies may fail to be merged due to the presence of independently assembled allelic contigs (haplotig) exhibiting significant structural and sequence variation (Roach et al., 2018; Formenti et al., 2019).

In this study, we generated the first Nanopore long read data (∼ 30× coverage) for the blacklip abalone (**Figure 1A**) and applied the hybrid meta-assembly approach to assemble its genome. Although the inclusion of long read generally improved the abalone genome contiguity and completeness, several haplotigs remain in the meta-assembly, leading to a larger assembly size and a higher representation of duplicated single-copy genes. We overcome this by performing a haplotig removal step, generating the first high-quality genome assembly for an Australian abalone. To demonstrate the utility of this resource, we annotated the genome and explored the genome for heat-shock proteins (HSPs), a highly conserved protein family whose synthesis has been previously linked to thermal stress in *Haliotis* species (Farcy et al., 2007; Li et al., 2012; Shiel et al., 2015).

## Materials and Methods

### Sample Collection and Nucleic Acid Extraction

Sample DU_JTF1, an offspring from brother/sister mating, was collected in 2017 from Jade Tiger Abalone Farm (Indented Head, Victoria, Australia) and stored frozen at −20°. Sample DU_PF1 is a captive bred specimen and was freshly sourced from Southern Ocean Mariculture (Port Fairy, Victoria, Australia) in 2018. Immediately after euthanization, its liver/gonad tissue was dissected and homogenized in DNA/RNA shield (Zymo Research, Irvine, CA).

For DU_JTF1, genomic DNA was extracted from multiple muscle biopsies (50 mg wet weight each) using a modified high salt SDS-lysis approach (Sokolov, 2000). For DU_PF1, the extraction of total RNA and DNA from the DNA/RNA shield preserved tissue used Zymo Quick RNA Miniprep kit and Zymo Quick DNA Miniprep kit (Zymo Research, Irvine, CA), respectively, according to the manufacturer’s instructions.

### Illumina Whole-Genome Sequencing

One hundred nanograms of gDNA from DU_JTF1 was sheared to 350 bp with a Q800 sonicator (Qsonica, Newtown, CT) and processed with NEB Ultra Library Preparation Kit (NEB, Ipswich, MA) according to the manufacturer’s instructions. The library was quantified on an Agilent 4200 TapeStation (Agilent, Santa Clara, CA) and sequenced on the Novaseq6000 (Illumina, San Diego, CA) located at the Deakin Genomics Centre using the run configuration of 2×150 bp.

### Illumina Transcriptome Sequencing

The integrity of total RNA extracted from DU_PF1 was assessed on an Agilent 4200 TapeStation (Agilent, Santa Clara, CA), after which, approximately 1 µg of the total RNA was processed using the Nugen Universal Plus mRNA-seq library preparation kit (NuGEN, Redwood City, CA). The 200-bp insert size library was sequenced on the same Novaseq6000 instrument as described above.

### Nanopore Sequencing

A total of 14 LSK-108 and five LSK-208 (obsolete) Nanopore libraries were prepared for sequencing (See **Supplemental Table 1** for details). Sequencing was performed on multiple MinION devices attached to a desktop running Ubuntu 16.04. Subsequent base-calling of the raw fast5 data to fastq files used Albacore v.2.3.3. The DU_JTF1 Nanopore libraries were prepared using 2 µg of Bluepippin size-selected template DNA (> 5kb). On the other hand, the DU_PF1 Nanopore libraries were prepared using 1 µg of column-purified DU_PF1 gDNA without size selection and sequenced on 3 MinION R9.4 flowcells.

### Pre-processing of Sequencing Reads and Genome Profiling

Illumina sequencing reads were processed with fastp v0.12.1 to remove low-quality bases (<Q20), as well as adapter and poly-G sequences at the 3′ end (Chen et al., 2018). Adapter-trimming and length filtering (>1,000 bp) of base called Nanopore reads used Porechop v0.2.4 (https://github.com/rrwick/Porechop), respectively. Prior to assembly, the processed Illumina reads were used to calculate kmer frequencies in Jellyfish2 v. 2.2.6 (Marçais and Kingsford, 2011). The kmer histogram for three different kmer length (k21, k25, and k31) were submitted to the Genomescope webserver for estimation of genome size, repeat content, and heterozygosity, based on a kmer-based statistical approach (Vurture et al., 2017).

### Genome Assembly

A total of six assemblies were generated from the data set as detailed in **Table 1**. Assembly A1 represents an Illumina-only assembly performed using Platanus v.1.2.4 (default setting), suited to highly heterozygous genomes (Kajitani et al., 2014). Assembly A2 was constructed from Nanopore long read using wtdbg v1.2.8 (https://github.com/fantasticair/wtdbg-1.2.8) (Ruan and Li, 2019) followed by two rounds of Racon v1.2.0 polishing (Minimap2-aligned Nanopore reads) and one round of Pilon v1.21 polishing (BWA-aligned Illumina reads) (Li, 2013; Walker et al., 2014; Vaser et al., 2017). Assembly A3 represents a DBG2OLC hybrid assembly combining Illumina (Platanus-assembled contigs) and Nanopore data (Ye et al., 2016). The correction of DBG2OLC assembly was performed as similarly described for the assembly A2. Subsequent merging (meta-assembly) of assembly A2 (long-read only assembly) and A3 (hybrid assembly) used QuickMerge v0.2 (Chakraborty et al., 2016) with the setting “-hco 5.0 -c 1.5 -l 330,000 -ml 5,000,” generating assembly A4. RNA-sequencing data generated from DU_PF1 was aligned to assembly A4 with HiSat2 v2.1.0 (Kim et al., 2015) followed by transcriptome-guided genome scaffolding with L_RNA_P (Zhu et al., 2018) to produce assembly A5 (**Table 1**).

**Table 1:** Blacklip ablone genome assembly and annotation statistics.

Parameter	Details			
Genome Annotation				
Assembly Name: Method description	Total Length (bp)	Number of Scaffolds	N50 (bp)	N90 (bp)
Organism		*Haliotis rubra* (blacklip abalone)		
Isolate		DU_JTF1 (inbred, brother/sister mating)		
	DU_PF1 (captive bred)			
Bioproject		PRJNA489521		
Biosample		SAMN09981888 (isolate DU_JTF1)		
		SAMN09981889 (isolate DU_PF1)		
GenBank assembly accession		GCA_003918875.1 (QKJH01)		
Assembled Length		1,378,265,264 bp		
Scaffold N_50_		1,227,833 bp		
Number of scaffolds		2,854		
GC content		40.52%		
BUSCO completeness Metazoa odb9 (n = 978)		91.6% Single-copy, 3% Duplicated, 1.6% Fragmented, 3.8% Missing		
Number of predicted transcripts		47,928		
Number of protein-coding genes		44,137		
Number of proteins				
with InterProScan annotation		24,743 (56%) 16,408 (37%) 6 (176 bp) 5 (2,001 bp)		
with Gene Ontology (GO) terms				
Average number (length) of exon per gene				
Average number (length) of intron per gene				
A1: Platanus (Illumina read assembly)	1,441,198,393	419,307	15,704	1,602
A2: wtdbg v1.2.8 (Nanopore read assembly)	1,599,130,340	25,589	374,753	20,473
A3: DBG2OLC (hybrid assembly)	1,980,045,538	14,010	331,813	56,290
A4:QuickMerge (meta-assemblies of A2 and A3)	1,908,803,906	11,876	659,954	57,873
A5: L_RNA_P (scaffolding of A4 with RNA-seq)	1,908,833,106	11,584	705,847	59,076
A6:PurgeHaplotigs (A5 haplotig removal)	1,378,265,264	2,854	1,227,833	232,104

### Removal of Redundant Contigs

Illumina reads were aligned to assembly A5 using bwa-mem v0.7.17 (Li, 2013). A read depth histogram (**Supplemental Figure 1**) was generated from this alignment in PurgeHaplotig v.1.0.2 to obtain the read depth cut off values required to identify haploid (redundant) contigs for subsequent reassignment or purging (Roach et al., 2018). To improve the handling of repetitive regions during haplotig purging, we also included a BED-format file containing the location of repetitive regions that was generated using Red, a machine learning-based *de novo* repeat detection tool (Girgis, 2015). The PurgeHaplotig-curated primary contigs representing the deduplicated primary haploid assembly was designated as assembly A6 and submitted to NCBI.

### Genome Annotation

Transcriptome reads were mapped with STAR v2.7.1 to assembly A6. The transcriptome alignment in BAM format and the soft-masked assembly A6 fasta file were used as the input for Braker v.2.1.2 (Hoff et al., 2015) that fully automate the training of gene prediction tools, GeneMark-ET (Lomsadze et al., 2014) and AUGUSTUS (Stanke et al., 2006). The predicted proteins sequences from Braker2 were functionally annotated using InterProScan v5 (Jones et al., 2014). The number and length of transcripts, exons, and introns were inferred from the genome annotation gff3 file that was similarly generated by Braker2.

### Comparative Genomics

The genome sequences and predicted proteins of red abalone (*Haliotis rufescens*), Pacific abalone (*H. discus hannai*), and owl limpet (*Lottia gigantea*) were obtained from their respective data repository for comparison with the newly sequenced blacklip abalone. Each genome assembly was assessed for completeness with BUSCOv3 based on the metazoa odb9 lineage dataset (Waterhouse et al., 2017). Then, orthologous groups of proteins were identified between *H. rufescens, H. discus hannai, H. rubra,* and *L. gigantea* using an all-vs-all DIAMOND blastp search and Markov Cluster (MCL) clustering approach as implemented in OrthoFinder v2.2.7 (default setting). The computation and visualization of ortholog intersections across the four marine gastropods used UpSetR (Conway et al., 2017).

### Identification of Heat Shock Protein 70 (HSP70)

The InterProScan annotation of *H. rubra* proteins was filtered for proteins containing the InterPro domain IPR013126 (Heat shock protein 70 family). The putative *H. rubra* heat shock proteins and their respective orthologs from *H. rufescens, H. discus hannai*, and *L. gigantea* identified by OrthoFinder2 were submitted to the GUIDANCE2 web server (Sela et al., 2015) for the detection and removal of unreliable sequences and alignment regions. IQTree v.1.6.5 (Nguyen et al., 2014) was used to construct a maximum likelihood tree based on the GUIDANCE2 alignment output followed by visualization in FigTree v 1.4.3 (https://github.com/rambaut/figtree/).

## Preliminary Analysis

Using the k-mer approach based on 291.7 Gb of Illumina short read data, the *H. rubra* haploid genome size was predicted to be between 1.24 to 1.31 Gb with moderate-high heterozygosity of 1.27 to 1.44% (**Supplemental Table 2**). Additional processing of the Illumina reads followed by another GenomeScope analysis with the max kmer coverage filter disabled resulted in a predicted haploid genome size of 1.56 Gb, a value closer to the 1.65 Gb haploid size estimated for a different haliotid species based on k-mer distributions (Nam et al., 2017; Masonbrink et al., 2019). Nanopore sequencing generated a total of 28 Gb (0.5-3.1 Gb *per* flowcell, more than 10 kb median read length) and 25 Gb data (6.8-11 Gb *per* flowcell, 1-2 kb median read length) for the DU_JTF1 and DU_PF1 libraries, respectively (**Supplemental Table 1**).

As expected, *de novo* assembly using only short reads resulted in a highly fragmented assembly contained in 419,307 scaffolds with an N_50_ of 15,704 bp. On the contrary, Nanopore long-read only assembly followed by polishing with Illumina data generated a significantly more contiguous assembly with an N_50_ of 374,753 bp with reduced fragmented BUSCO genes (assembly A2 in **Figure 1B**). A similar N_50_ length was observed for the DBG2OLC hybrid assembly albeit with a marked increase in the assembled genome length (**Table 1**) and the number of duplicated single-copy BUSCO genes (assembly A3 in **Figure 1B**). After the merging of assemblies A2 and A3 using QuickMerge, we observed a 200-kb increase in the N_50_ length and a slight decrease in assembled genome size (**Table 1** and assembly A4 in **Figure 1B**). Another slight gain of 45.8 kb in N_50_ length was observed with the transcriptome-guided scaffolding (assembly A5 in **Figure 1B**).

Consistent with the high genome heterozygosity as estimated by GenomeScope (**Supplemental Table 1**), a substantial portion of assembly A5 was still represented by redundant scaffolds reflecting distinct haplotypes from the heterozygous genomic region as evidenced by the presence of a secondary peak representing allelic contigs in the read coverage frequency plot (**Supplemental Figure 1**). After processing with Purge Haplotigs, the final *H. rubra* haploid assembly was contained in 2,854 scaffolds with an N_50_ of 1.23 Mb. Compared with assembly A5, this represents a 75.36% decrease and 73.95% increase in the number of scaffolds and N_50_ length, respectively. The removal of redundant contigs also resulted in a genome assembly with lower duplicated gene content (**Figure 1B**).

A total of 44,137 protein-coding genes were predicted from the current genome assembly and training model, of which 24,743 were functionally annotated by InterProScan. A total of 22,180 orthologous groups were identified across the four marine gastropod genomes, of which 6,652 were core ortholog clusters, and 3,689 were exclusively shared by the currently sequenced *Haliotis* species (**Figure 1C**). Although *L. gigantea* is more divergent to *H. rufescens* and *H. rubra* compared with *H. discus hannai*, it shared 3,737 private ortholog clusters with *H. rubra* and *H. rufescens*. It is plausible that the training model used by Nam et al. (2017) may have under-predicted the protein-coding genes in the *H. discus hannai* genome since its BUSCO genome completeness is comparable to that of *H. rufescens* and *H. rubra* (**Figure 1C**).

Given the current threats of extreme temperature events, and rising sea surface temperatures in south-eastern Australia (Oliver et al., 2017), the identification of candidate genes associated with thermal stress, and variants influencing their expression, is important for fisheries management and conservation purposes. We identified seven putative HSP70 proteins from *H. rubra* (**Figure 1D**) that formed a strongly supported (ultrafast bootstrap support values > 95%) monophyletic clustering with their respective *Haliotis* spp. orthologs. Sister grouping of *H. discus hannai* and *H. rufescens* HSP70 orthologs with *H. rubra* occupying the basal position was observed in a majority of the haliotid HSP70 clades (**Figure 1D**).

## Future Avenues

The draft genome sequence presented in this study is the first for an Australian abalone species only the third *Haliotis* genome sequence to be made available in the public data repository. With less than 3,000 scaffolds and more than 90% of the genome contained in scaffolds larger than or equal to 200 kb (**Table 1**), this draft genome will serve as a suitable base reference for future Hi-C scaffolding and chromosomal-level genome assemblies. The substantial improvement in various assembly metrics after a quick and resource-efficient haplotig-purging step indicates that this can be an important step for generating highly contiguous hybrid assembly from heterozygous marine organisms. The annotated genome is expected to assist in future genomic breeding program by enabling the precise identification of candidate gene variants affecting ecologically and commercially important traits.

## Data Availability

Raw Illumina reads are available under the Bioproject PRJNA489521. Base-called Nanopore reads have been deposited in the Zenodo repository (https://doi.org/10.5281/zenodo.2602223). The Whole Genome Shotgun project has been deposited at DDBJ/EMBL/GenBank under accession number QKJH01. Genome annotation, BUSCO calculation, genome assemblies, and Orthofinder2 output have also been deposited in the Zenodo repository (http://doi.org/10.5281/zenodo.3320876).

## Author Contributions

HG, AM, and CA conceived the project. HG designed the experiments. AM, CS, LM, and MG collected the specimens. YW performed RNA extraction and transcriptome sequencing. HG performed DNA extraction and whole-genome sequencing. HG, MT, and JS conducted genome analysis and assembly. HG, AM, and CA drafted the manuscript. All authors edited and contributed to the article.

## Funding

Funding for this study was provided by the Deakin internal research grant scheme (SEBE-RGS-2018- 0509.31325.31.01) awarded to HG, AM, CS, and CA.

## Conflict of Interest Statement

MG was employed by Southern Ocean Mariculture and LM was employed by Jade Tigers Abalone. The remaining authors declare that the research was conducted in the absence of any commercial or financial relationships that could be construed as a potential conflict of interest.

## Supplementary Material

Supplemental Figure 1 Illumina read depth histogram of Assemblies A5 and A6

Supplemental Table 1 Nanopore sequencing details and data output

Supplemental Table 2 Alignment-free genome profiling with Illumina reads
