# Supplementary figures and images for: Best Foot Forward: Nanopore Long Reads, Hybrid Meta-Assembly, and Haplotig Purging Optimizes the First Genome Assembly for the Southern Hemisphere Blacklip Abalone (Haliotis rubra)
Source: Front Genet. 2019 Sep 25;10:889. doi: 10.3389/fgene.2019.00889 (PMC6774278; doi:10.3389/fgene.2019.00889)

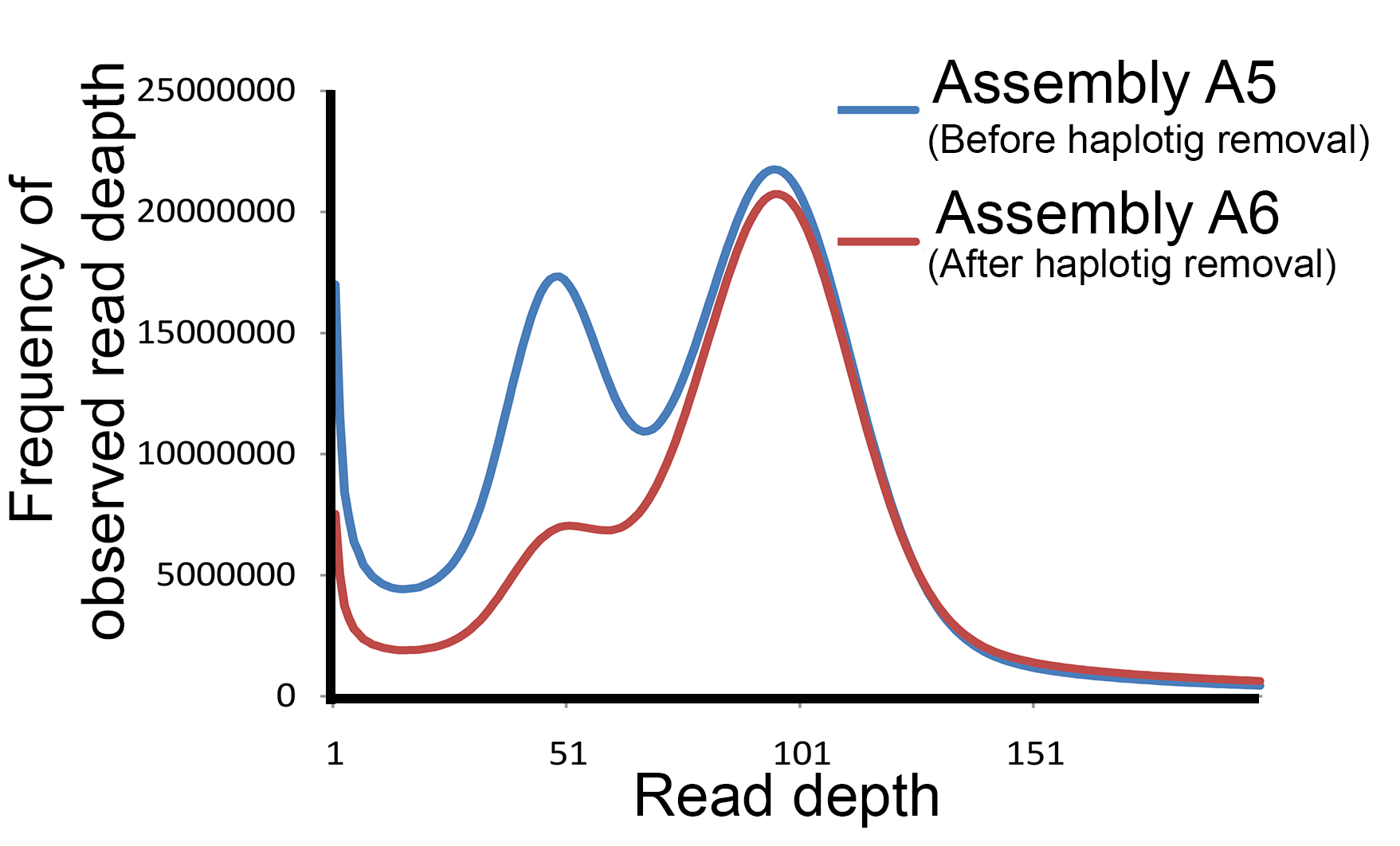

Supplement: Supplemental Figure 1 — Illumina read depth histogram of Assemblies A5 and A6 [file Image_1.tif]
